# *CYP3A5* Gene-Guided Tacrolimus Treatment of Living-Donor Egyptian Kidney Transplanted Patients

**DOI:** 10.3389/fphar.2020.01218

**Published:** 2020-08-11

**Authors:** Effrosyni Mendrinou, Mohamed Elsayed Mashaly, Amir Mohamed Al Okily, Mohamed Elsayed Mohamed, Ayman Fathi Refaie, Essam Mahmoud Elsawy, Hazem Hamed Saleh, Hussein Sheashaa, George P. Patrinos

**Affiliations:** ^1^ Department of Pharmacy, School of Health Sciences, University of Patras, Patras, Greece; ^2^ The Urology-Nephrology Center, Department of Dialysis and Transplantation, Mansoura University, Mansoura, Egypt; ^3^ Department of Nephrology, Zagazig University, Zagazig, Egypt; ^4^ Urology and Nephrology Center, Department of Laboratories, Mansoura University, Mansoura, Egypt; ^5^ Zayed Center of Health Sciences, United Arab Emirates University, Al-Ain, United Arab Emirates; ^6^ Department of Pathology, College of Medicine and Health Sciences, United Arab Emirates University, Al-Ain, United Arab Emirates

**Keywords:** CYP3A5, kidney transplantation, living donor, tacrolimus, Egyptian population, dose requirements, C/D ratio, tacrolimus blood levels

## Abstract

**Background:**

Tacrolimus is an approved first-line immunosuppressive agent for kidney transplantations. Part of interindividual and interethnic differences in the response of patients to tacrolimus is attributed to polymorphisms at CYP3A5 metabolic enzyme. *CYP3A5* gene expression status is associated with tacrolimus dose requirement in renal transplant recipients.

**Materials and Methods:**

In this study, we determined the allelic frequency of CYP3A5*3 in 76 renal transplanted patients of Egyptian descent. Secondly, we evaluated the influence of the *CYP3A5* gene variant on tacrolimus doses required for these patients as well on dose-adjusted tacrolimus trough-concentrations.

**Results:**

The CYP3A5*3 variant was the most frequent allele detected at 85.53%. Additionally, our results showed that, mean tacrolimus daily requirements for heterozygous patients (CYP3A5*1/*3) were significantly higher compared to homozygous patients (CYP3A5*3/*3) during the first year after kidney transplantation.

**Conclusion:**

This is the first study in Egypt contributing to the individualization of tacrolimus dosing in Egyptian patients, informed by the *CYP3A5* genotype.

## Introduction

Chronic Kidney Disease (CKD) is a long-term, progressive, and irreversible condition characterized by functional and structural kidney damages lasting for at least 3 months (Levin et al., 2013; Webster et al., 2017). Kidney transplantation is the optimal kidney replacement therapy for patients who have reached end-stage renal disease (ESRD) (Thongprayoon et al., 2020). Transplant recipients require life-long immunosuppression to prevent allograft rejection. Tacrolimus, a calcineurin inhibitor, is the most frequently used drug in kidney transplantation recipients. The impressive results of tacrolimus treatment, however, are offset by its side effects, narrow therapeutic index and variable and unpredictable pharmacokinetics (Tang et al., 2016). For this reason, therapeutic drug monitoring (TDM) is crucial in daily practice. Renal transplant recipients usually receive standard weight-based dose which is then adjusted according to TDM to maintain tacrolimus blood concentrations within the therapeutic range. However, using TDM do not guarantee optimal treatment efficacy or lack of rejections and adverse reactions (Birdwell et al., 2015; Yanik et al., 2019). Genetic factors are considered to play important role in the interindividual and interethnic variability in pharmacokinetics of tacrolimus (Ghafari et al., 2019).

CYP3A5 is an enzyme responsible for the metabolism of tacrolimus. Single nucleotide polymorphisms in *CYP3A5* gene explain 40–50% of the variability in tacrolimus metabolism and clearance (Woillard et al., 2017). The A to G transition at position 6986 in intron 3 of the *CYP3A5* gene is the most well-studied genomic variant which contributes to dose requirement of tacrolimus (Prasad et al., 2020). CYP3A5*3 allele results in alternative spicing of the mRNA which leads to absence of CYP3A5 protein activity and is associated with reduced tacrolimus dose requirement (Ferraris et al., 2011). The presence of the wild-type allele (CYP3A5*1) contributes significantly to the increase of CYP3A activity associated with recovery of renal function after transplantation (Suzuki et al., 2015). Two more variant alleles, CYP3A5*6 and CYP3A5*7, result also at loss of expression of the functional protein in homozygotes (Birdwell et al., 2015).

Several studies in different populations have shown that *CYP3A5* expressors, who carry at least one CYP3A5*1 allele require 50% (1.5–2-fold) higher tacrolimus doses compared to *CYP3A5* non-expressors those who are homozygous for the variant alleles (CYP3A5*3, CYP3A5*6, or CYP3A5*7) (Birdwell et al., 2015; Chen and Prasad, 2018). However, this association between *CYP3A5* genotypes and tacrolimus dose requirement has not yet been studied in Egyptian kidney transplantation recipients.

In this study, we aimed to determine the allelic frequency of CYP3A5*3 among Egyptian patients that have undergone transplantation and to evaluate the influence of this polymorphism on tacrolimus daily dose and on metabolism rate in adult patients during the first year after kidney transplantation.

## Materials and Methods

### Study Population

For the present study, 76 unrelated kidney transplanted adult patients were enrolled in Urology and Nephrology Center at Mansoura University Hospital in Egypt. All patients underwent renal transplantation from living donors and were under tacrolimus immunosuppressive treatment for at least one year. Recipients received a standard bodyweight-based tacrolimus initial dose (day -1 before transplantation) of 0.1 mg/kg twice per day. Blood samples were collected into EDTA tubes and stored at -80°C till analyzed. Therapeutic drug monitoring was applied to all samples for dose adjustment. The target whole-blood concentration in early period after transplantation is 10–20 ng/ml and in the maintenance period (after 3 months) 5–10 ng/ml. Tacrolimus daily dose, tacrolimus blood levels, demographic, and clinical data were obtained from medical files of the patients at the beginning of the post-transplant period and at 12 months after transplantation. Patients with diarrhea or vomiting, liver disease, advanced renal dysfunction, or other disorders that could have altered the absorption of tacrolimus or patients that will be co-prescribed drugs that affect the pharmacokinetics of tacrolimus and its pharmacological effect (antifungals, antiepileptics, macrolide antibiotics) were excluded from the study.

The study was conducted in compliance with the declaration of Helsinki and was approved by the Ethics Committee of the Mansoura University Hospital and written informed consent was obtained from all subjects.

### DNA Extraction and Genotyping

Total genomic DNA was extracted from the peripheral blood, followed by determination of its concentration and purity. The *CYP3A5* single nucleotide polymorphism (SNP) – CYP3A5*3 (rs776746) was genotyped by PCR-restriction fragment length polymorphism (RFLP), using the SspI restriction endonuclease as previously described (Mendrinou et al., 2015).

### Statistical Analysis

Estimation of allele and genotype frequencies was performed using gene counting method and their deviation from Hardy-Weinberg equilibrium was assessed by Pearson’s goodness of fit chi-square test (degree of freedom = 1). Continuous variables are shown as mean and standard deviation and qualitative data are expressed as frequency and percentage.

Continuous data were tested for normality using Kolmogorov-Smirnov and Shapiro-Wilk tests (p = 0.05) and visualized with Q-Q plots. Depending on the distribution, comparisons for variables between two groups were performed with two-tailed test or Wilcoxon test for related samples and with unpaired t-test or Mann-Whitney test for independent samples. The categorical data were analyzed using two-tailed Fisher’s exact test.

In the present study patients were divided into two groups according to their genotype [CYP3A5 expressors (*1/*1 or *1/*3) and CYP3A5 non-expressors (*3/*3)]. Both groups were examined for statistically significant difference in dose requirements, tacrolimus blood levels, and C/D ratio (dose corrected trough concentration of Tac). These data were compared at different time points among related samples (patients with the same genotype) and at the same time points among independent samples (patients with different genotype).

Statistical analysis was performed using SPSS Statistics 25.0 (IBM SPSS software) and GraphPad Prism 8.0. The significance level was set at p<0.05.

## Results

### Demographic Characteristics of the Patients

A total of 76 kidney transplant recipients were included in this study and they all were adults and self-reported Egyptians. According to the date of the transplantation, there were missing data for 17 of the patients regarding tacrolimus dose. The characteristics of 59 recipients according to their CYP3A5 genotype are shown in **Table 1**. There were no statistically significant differences between the two groups with respect to sex, family history, age of CKD, age at transplantation, time waiting for transplantation, incidence rejection, or donor type.

**Table 1:** Comparison of the clinical characteristics, tacrolimus daily dose, tacrolimus blood levels, and C/D ratio of the study population between *CYP3A5* expressors and non-expressors.

Characteristics	Non-expressors (*3/*3) n = 41	Expressors (*1/*3, *1/*1) n = 18	P value
Gender, n (%)			
Male	35 (85.37%)	13 (72.2%)	0.2841
Female	6 (14.63%)	5 (27.8%)	
Onset of CKD, years, mean (range) (SD)	27.2 (9–55)	31.2 (14–65)	0.2403
Onset at transplantation, years, mean (range) (SD)	29.2 (10–55)	32.8 (14–67)	0.2983
Time waiting for transplant, years, mean (range) (SD)	2 (0–6)	1.56 (0–4)	0.1965
Graft rejection, n (%)			
Yes	8 (19.5%)	6 (33.3%)	0.3224
No	33 (80.5%)	12 (66.7%)	
Family history, n (%)			
Yes	3 (7.3%)	2 (11.1%)	0.6359
No	38 (92.7%)	16 (88.9%)	
Donor type, n (%)			
Living Related	33 (80.5%)	14 (77.8%)	1.0000
Living unrelated	8 (19.5%)	4 (22.2%)	
Initial Tac D, mg/day, mean (range) (SD)	6.76 (2–11)	9.86 (6–14)	**<0.0001**
1-year Tac D, mg/day, mean (range) (SD)	4.21 (1.5–10.5)	7.81 (2.5–13)	**<0.0001**
Initial Tac C, ng/ml, mean (range) (SD)	7.09 (2–22.6)	5.89 (2–13.5)	0.3035
1-year Tac C, ng/mL, mean (range) (SD)	7.39 (3.3–11.7)	7.15 (4.9–9.9)	0.6373
Initial C/D ratio, ng/ml per mg/day, mean (range) (SD)	1.50 (0.2–9.4)	0.64 (0.18–1.5)	0.0586
1-year C/D ratio, ng/ml per mg/day, mean (range) (SD)	2.10 (0.6–5.8)	1.10 (0.63–2.84)	**0.0003**

D, tacrolimus daily dose; C, tacrolimus blood concentration; SD, standard deviation.

Bolded data are those which are statistically significant.

### Frequency of the CYP3A5*3 Variant in Kidney Transplant Recipients

**Figure 1:**
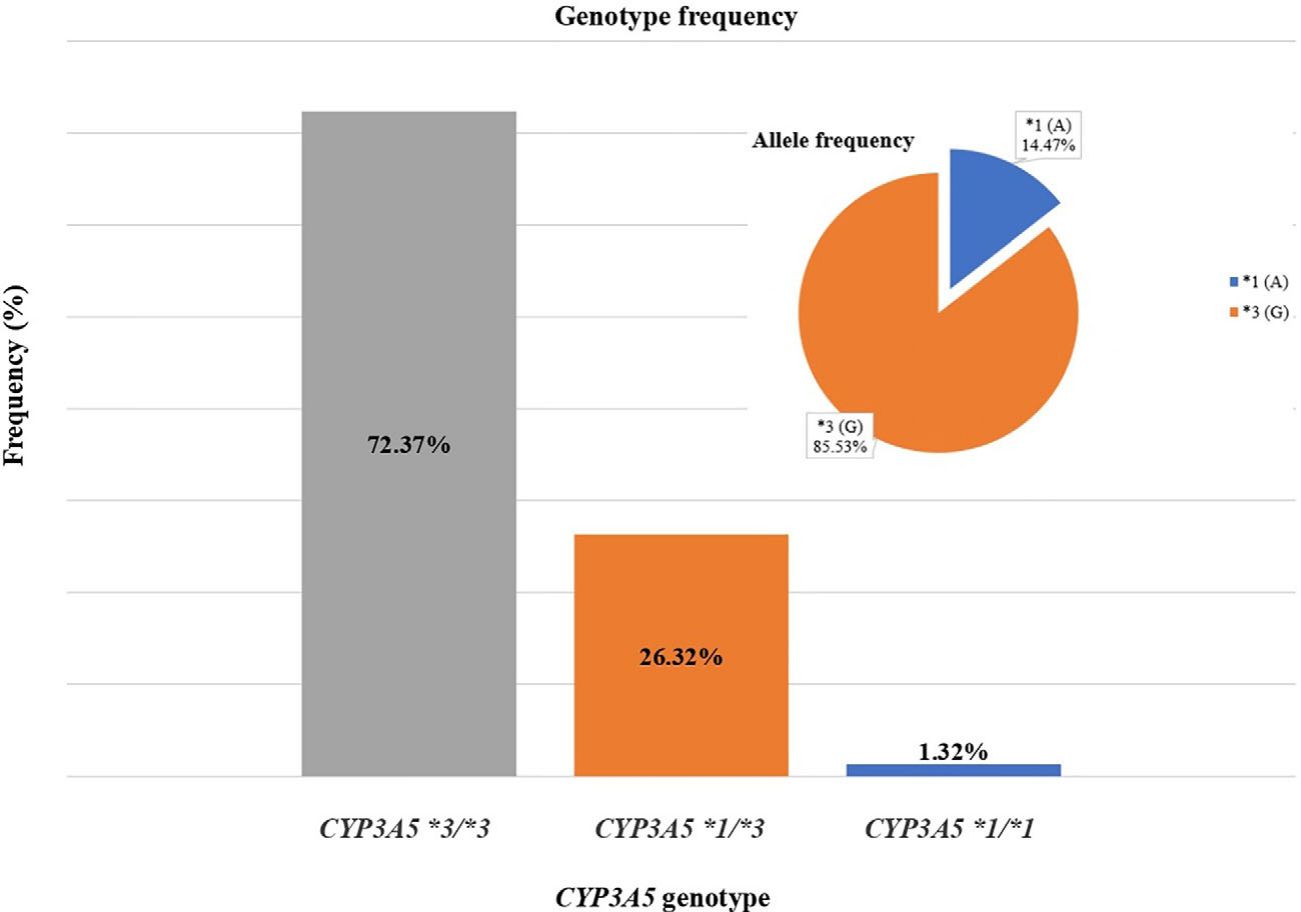
Genotype and allelic frequencies of 76 renal transplant recipients for *CYP3A5* gene.

Of the 76 kidney transplant recipients, the CYP3A5*3/*3 genotype was observed in 55 (72.37%) cases, CYP3A5*1/*3 in 20 (26.32%) cases, and CYP3A5*1/*1 in 1 (1.32%) case. Total allelic frequency was 85.53% for CYP3A5*3 and 14.47% for CYP3A5*1 (**Figure 1**). No deviation from Hardy-Weinberg equilibrium was observed for the genotype frequencies (χ^2^ = 0.58323 < 3.841).

### Association of the *CYP3A5* Genotype With Tacrolimus Dose, Tacrolimus Blood Levels, and C/D Ratio

**Figure 2:**
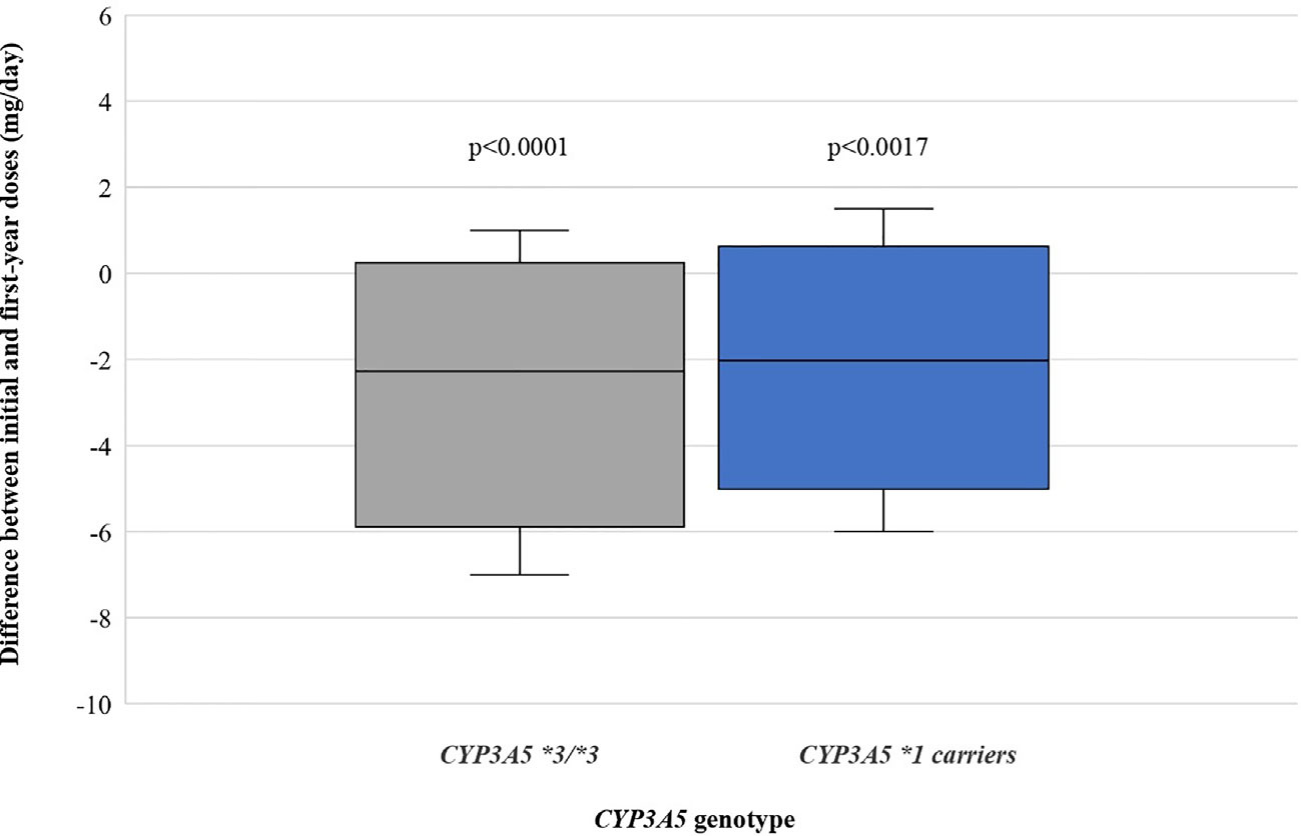
Differences between initial and first-year doses as stratified by *CYP3A5* genotype.

For the 59 patients, tacrolimus initial doses (mean ± standard deviation) for CYP3A5*1 carriers and CYP3A5*3/*3 groups were 9.861 ± 2.182 (range: 6.0–14.0) and 6.756 ± 2.478 mg/day (range: 2.0–11.0), while doses one year after transplantation were 7.806 ± 3.158 (range: 2.5–13.0) and 4.207 ± 2.083 mg/day (range: 1.5–10.5), respectively. This shows a significant reduction of the dosage for both genotypic groups, 20.84% for expressors (CYP3A5*1/*3 or *1/*1) (P = 0.0017) and 37.73% for non-expressors (CYP3A5*3/*3) (P < 0.0001). Differences between initial and first-year doses are shown in **Figure 2**.

**Figure 3:**
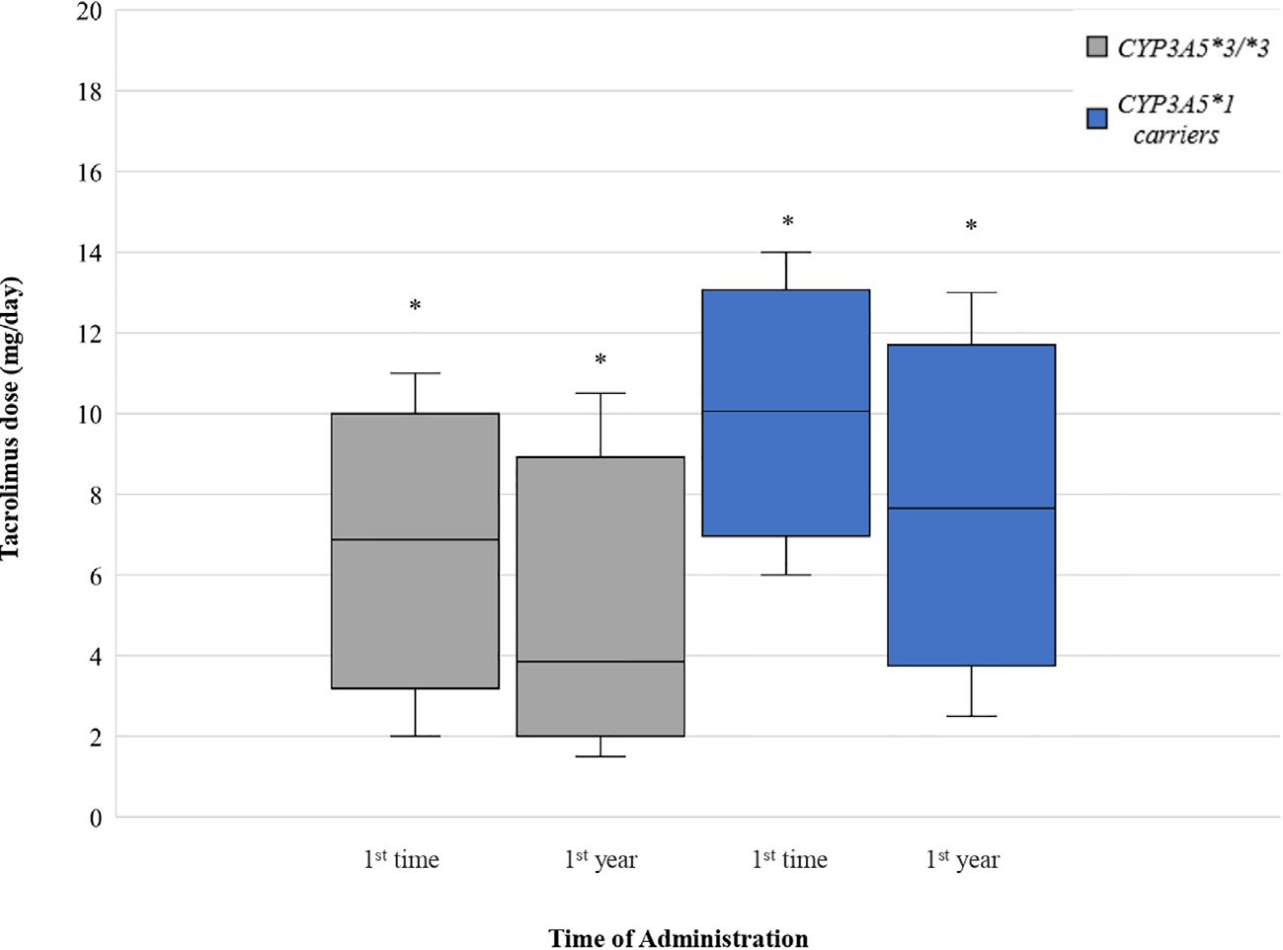
Tacrolimus dose for *CYP3A5* genotypes. Each genotype appears as paired blots (first blot for initial dose-second blot for first-year dose). *p < 0.05.

Comparing the starting daily dose between CYP3A5*3/*3 and CYP3A5*1 carriers, mean dose for CYP3A5*1 carriers was significantly higher (45.96%) than for CYP3A5*3/*3 (P < 0.0001). One-year mean tacrolimus dose for CYP3A5*1 carriers was 85.55% higher than for CYP3A5*3/*3 (P < 0.0001) (**Figure 3**).

Average tacrolimus blood concentrations in *CYP3A5* non-expressors was higher in both time points compared with *CYP3A5* expressors. However, there was no significant differences between the two groups neither at the early post-transplant period (p = 0.3035) nor at the maintenance period (p = 0.6373).

**Figure 4:**
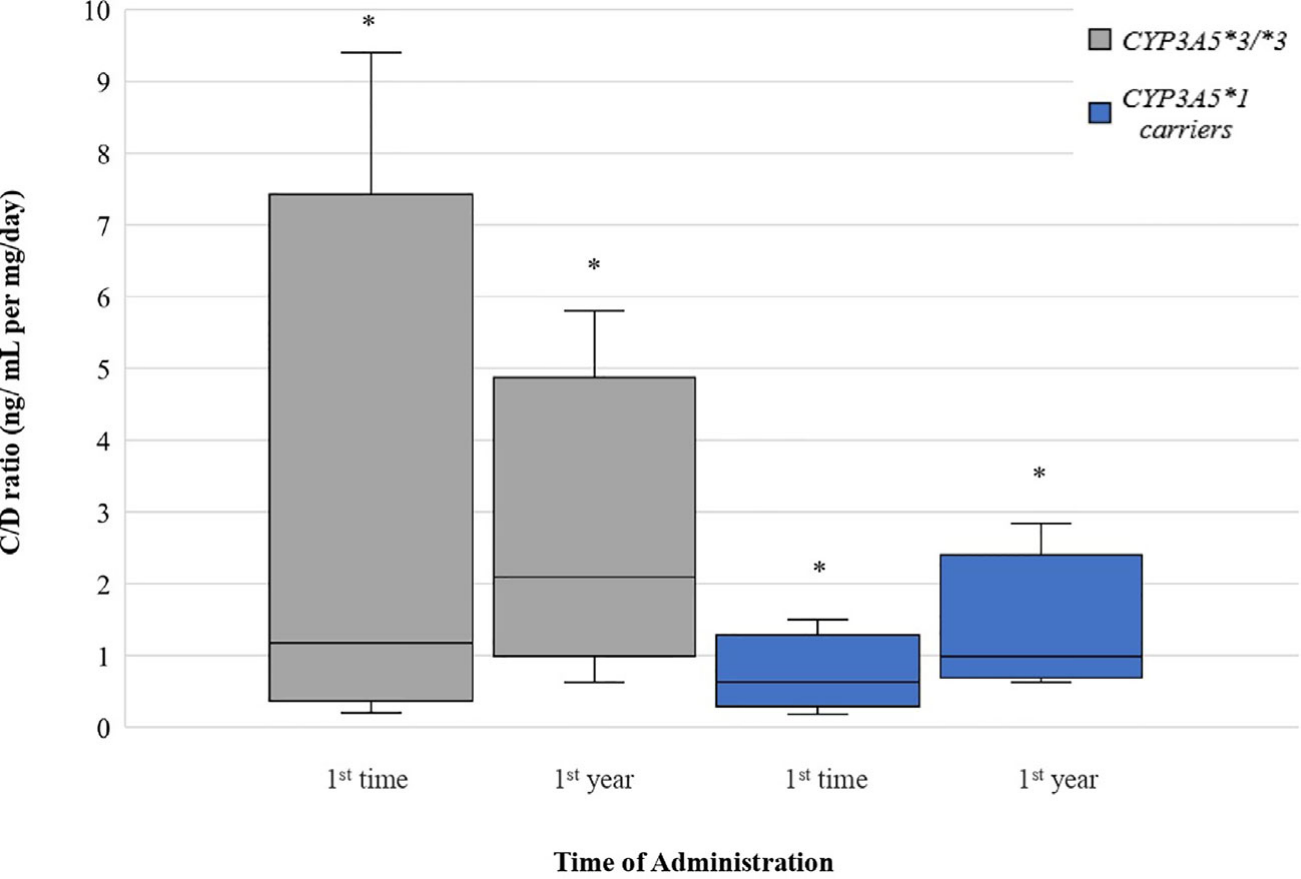
Dose-Adjusted Tacrolimus Trough-Concentrations for *CYP3A5* genotypes. Each genotype appears as paired blots (first blot for initial dose-second blot for first-year dose). *p < 0.05.

CYP3A5*1 recipients exhibited significantly lower C/D ratios (47.89% lower) than those homozygous for the variant allele (*3/*3) at one year of treatment (1.097 ± 0.5829 and 2.105 ± 1.030 ng/ml per mg/day, respectively, p = 0.0003). However, there was no significant difference between the two groups at the early post-transplant period (p = 0.0586). Significant increase was observed at C/D ratios comparing the two time points among CYP3A5*1 carriers (p = 0.0003) and among CYP3A5*3/*3 recipients (p = 0.0123) **(** **Figure 4**).

## Discussion

The biggest challenge for clinicians is the long-term maintenance of renal grafts after a kidney transplantation. Tacrolimus is one of the currently used immunosuppressive therapies, but its administration may be the causative factor of many side effects and graft rejection (Thishya et al., 2018). In addition to the highly variable oral bioavailability, pharmacokinetics of tacrolimus is characterized by diversity among individuals in the first-pass metabolism and systemic clearance. These differences are largely due to *CYP3A5* polymorphisms and their effect on the metabolism of tacrolimus.

Pharmacogenomics studies have reported significant association between the *CYP3A5* genotype and the daily doses required for kidney transplant recipients. Most of them noticed that tacrolimus doses were significantly higher in patients carrying *1 allele (CYP3A5*1/*1 + CYP3A5*1/*3) compared to recipients homozygous for *3 allele (CYP3A5*3/*3) (Tang et al., 2016). Our study aimed to analyze the distribution of *CYP3A5* allele frequency in the Egyptian population. In the study population (n = 76) the three genotypic groups, CYP3A5*1/*1, CYP3A5*1/*3, and CYP3A5*3/*3 were observed in 1.32, 26.32, and 72.37% respectively. The distribution of *CYP3A5* gene showed that the CYP3A5*3 allele was 85.53%. In previous studies in the Egyptian population, different frequencies were reported for the CYP3A5*3 allele, ranging from as low as 11% to as high as 78% (Zayed and Mehaney, 2015; Abo El Fotoh et al., 2016; El Wahab et al., 2017). Studies published in other North African populations (Algerians, Morocco, Tunisians, Libyans) showed that the CYP3A5*3 allele was the most prevalent with a frequency that reaches even 90% (Novillo et al., 2015; Fernández-Santander et al., 2016), whereas in the African population as a whole is observed great diversity from 4 to 95% (Zhou et al., 2017).

Several studies have been conducted in North Africans in order to evaluate the effect of *CYP3A5* variants on tacrolimus dosage and on tacrolimus blood concentrations normalized by the dose and proved that there is significant difference between renal transplant patients with the CYP3A5*1 allele compared to homozygotes for the CYP3A5*3 allele, especially during the early post-transplant phase (Elmachad et al., 2012; Aouam et al., 2015). To our knowledge, this is the first study to examine the association of the CYP3A5*3 allele with tacrolimus dose requirements and C/D ratios in Egyptian kidney transplant recipients. To date, in the Egyptian population, some studies have been conducted examining the correlation of the *CYP3A5* genotype but in liver transplant patients (Fathy et al., 2016; Helal et al., 2017). Our results showed that tacrolimus doses were reduced between the first administration and one year after transplantation, regardless of genotype. Additionally, individuals homozygous for the CYP3A5*3 allele need significantly lower tacrolimus daily dose than those carrying *1 allele (p < 0.05). Concentration/dose ratio was significantly lower in CYP3A5*1 expressors. All these indicate that *CYP3A5* expressors require a larger tacrolimus dose in order to maintain the same blood concentration.

Although there are minor limitations in our study, single center and small cohort, our results showed that frequency of the CYP3A5*3 variant seems to be higher as compared with previous studies in the Egyptian population and in agreement to that reported prevalence of this allele for other North African or Caucasian populations. Furthermore, comparison of tacrolimus dose requirement for renal transplant patients showed statistically significant difference among genotypes. It is important to draw up different treatment plan for different recipients. As *CYP3A5* shows great heterogeneity in African population, there is a need for pharmacogenomic testing prior to tacrolimus administration after kidney transplantation to achieve genotype-guided dose and contribute to a better-individualized immunosuppressive therapy.

## Data Availability Statement

The raw data supporting the conclusions of this article will be made available by the authors, without undue reservation, to any qualified researcher.

## Ethics Statement

The studies involving human participants were reviewed and approved by Mansoura University Hospital. The patients/participants provided their written informed consent to participate in this study.

## Author Contributions

EM, MMa, and GP conceded the study. MMa, AA, MMo, AR, EE, HHS, and HS provided samples and clinical data. EM performed the analysis. EM and GP compiled the draft manuscript. GP and HS provided funding. All authors contributed to the article and approved the submitted version.

## Conflict of Interest

The authors declare that the research was conducted in the absence of any commercial or financial relationships that could be construed as a potential conflict of interest.
